# Correlation of differential expression of MEG3 lnc-RNA with biochemical parameters in Type-II diabetic patients with and without diabetic kidney disease

**DOI:** 10.12669/pjms.42.1.12591

**Published:** 2026-01

**Authors:** Huma Saeed Khan, Uzma Zafar, Waqar Ahmad, Saba Khaliq

**Affiliations:** 1 Dr. Huma Saeed Khan, Department of Physiology, University of Health Sciences, Lahore, Pakistan; 2 Dr. Uzma Zafar, Department of Physiology, University of Health Sciences, Lahore, Pakistan; 3 Dr. Waqar Ahmed, Department of Nephrology, Sheikh Zayed Hospital, Lahore, Pakistan; 4 Dr. Saba Khaliq, Institute of Allied Health Sciences, University of Health Sciences, Lahore, Pakistan

**Keywords:** Diabetes Mellitus, Diabetic kidney disease, Diabetic complications, MEG3 lncRNA

## Abstract

**Background & Objectives:**

Diabetic kidney disease (DKD) is a complication of type-II diabetes mellitus (DM) leading to significant morbidity and mortality. Long non-coding RNAs (lnc-RNAs) are a class of ribonucleic acid that exert their effects in development of such complications through epigenetic modifications. The study was conducted with the objectives to compare the relative expression of MEG3 lnc-RNA in patients of Type-II DM, with and without DKD, and to determine the correlation of this expression with blood and urine biomarkers.

**Methodology:**

A cross-sectional study was conducted at the University of Health Sciences, Lahore, after ethical approval from December 2022 till October 2023. Study participants were recruited in three groups having DKD, Type-II DM and healthy individuals, each group having 68 participants. Analysis of blood and urine biomarkers was performed. MEG3 lnc-RNA expression was determined by using RT-PCR. Kruskal Wallis test, post hoc Mann-Whitney U test and Spearman’s correlation were used for data analysis.

**Results:**

The relative expression of MEG3 lnc-RNA was significantly higher in the DKD group (median=3.83, IQR=1.22-3.83) as compared to the DM (median= 2.22, IQR= 0.49-3.12) and healthy groups (median= 1.97, IQR= 1.17-1.97) with a *p-*value of 0.002. There was a significant positive correlation between MEG3 expression and fasting insulin in the DKD group, a significant negative correlation with fasting insulin in the DM group, a significant negative correlation with serum very low-density lipoproteins in the healthy group, and a significant negative correlation between MEG3 lnc-RNA expression and urinary creatinine in the DKD group.

**Conclusions:**

MEG3 lnc-RNA has a higher relative expression in the patients suffering from DKD in the local diabetic population. In future, it may be used as a diagnostic marker as well as for inducing epigenetic modification through RNA transfection.

## INTRODUCTION

Diabetes Mellitus (DM) is a disease resulting from multiple endocrine and metabolic processes, often characterized by either an impairment in the secretion of insulin from the pancreatic beta cells, or by a lack of responsiveness of the target cells to the secreted insulin, or both.^1^ Over the last few decades, this disease has turned into a global problem, and latest statistics provided by the International Diabetes Federation are alarming, showing Pakistan at number 1 in the list of countries having a prevalence of 30.8% for diabetes when adjusted for age.^2^

Among the long-term complications of diabetes, Diabetic kidney disease (DKD) represents a major cause of morbidity and mortality and accounts for up to 47% of cases of end stage renal disease in developed countries and up to 60% of the cases in far eastern countries including Malaysia and Singapore. It is diagnosed clinically after establishing a diagnosis of diabetes, accompanied by a fall in glomerular filtration rate and urinary albumin loss.^3^ Recently it was estimated that 130/1000 cases of DM develop DKD.^4^ The disease involves structural and functional changes in the kidney, such as expansion of renal mesangial tissue, tubulointerstitial changes, glomerulosclerosis, inflammation, fibrosis and apoptosis due to metabolic and hemodynamic abnormalities.^5^ In the past ten years, genome-wide association studies (GWASs) including larger cohorts have proven to be a powerful approach for identifying genetic risk factors associated with diabetic kidney disease (DKD). Furthermore, epigenome-wide association studies are also being conducted to explore the role of DNA methylation in the disease.^6^

Maternally expressed gene 3 (MEG3), is found on chromosome position 14q32.3 in the DLK1-MEG3 locus. It was first reported in the literature as a novel gene in 2000 and is comprised of 10 exons. The RNA coded by this gene is an lnc-RNA, called MEG3 lnc-RNA. The length of this lnc-RNA is 1600 nt. It is a nuclear RNA which is functionally classified as a regulatory long noncoding RNA because of its ability to alter the p-53 dependent and independent pathways in carcinoma.^7^

The downregulation of MEG3 in animal models such as mice leads to a reduction in secretion of insulin from the pancreas and causes impaired glucose tolerance. The expression of this lnc-RNA is 20 times more in beta cells than in the alpha cells.^8^ MEG3 lnc-RNA contributes to vascular dysfunction by modulating the endothelial cells. It has been demonstrated that MEG3 knockdown in vitro can increase the phosphorylation of the phosphoinositide 3 kinase / AKT (PI3K/AKT) axis in the downstream insulin signaling pathways, and induce an increase in endothelial cell proliferation, migration, and abnormal angiogenesis in microvasculature such as the retina^9^ thus playing a role in microvascular complications. Human and animal studies have shown that MEG3 lnc-RNA can enhance cell fibrosis and inflammatory response in diabetic nephropathy.^10^

Studies reveal a contradictory role of MEG3 lnc-RNA over-expression, based on cell types or in the context of in vitro vs in vivo observations. Over expression of MEG3 lnc-RNA in cultured podocytes can attenuate high glucose mediated podocyte injury, cell migration and production of reactive oxygen species thus increasing the viability of podocytes.^11^ However, MEG3 lnc-RNA is expressed differentially by 4-fold in the serum of patients of diabetic nephropathy and MEG3 knockdown can induce apoptosis of mesangial cells in animal models.^12^ Epithelial to mesenchymal transition (EMT) is a hall mark in the pathogenesis of DKD, and a crucial role of MEG3 lnc-RNA has been demonstrated in the epigenetic modification of the amino terminals of the histones leading to transition of epithelial cells to mesenchymal cells in cultured oesophageal cancer cells.^13^

DKD is traditionally diagnosed by using routine clinical markers such as serum creatinine, urinary albumin and estimated GFR. However, these markers become detectable only after a significant amount of damage has already occurred in the renal tissue and the disease has progressed to advanced stages.^14^ There is evidence in published literature that lnc-RNAs can be utilized as potential targets for becoming markers for identification of disease and to determine the progression and prognosis of disease. Few can be targeted for therapeutic intervention.^15^ There is a relative deficiency of literature establishing the influence of long non-coding RNA in the pathogenesis of DKD in our population. This study aimed to measure and compare the expression of MEG3 lnc-RNA in peripheral blood mononuclear cells (PBMCs) of Type-II diabetic patients with and without diabetic kidney disease, and to evaluate its correlation with selected blood and urine biomarkers.

## METHODOLOGY

This cross-sectional study was performed in the Department of Physiology, University of Health Sciences, Lahore, and Department of Nephrology, Sheikh Zayed Hospital, Lahore, from December 2022 till October 2023.

### Ethical Approval:

It was obtained from the ethical boards of both institutions (letter no. UHS/Reg-21/ERC/6603; dated November 10, 2021 and SZMC/IRB/241/2022).

The sample size was determined by using the WHO sample size calculator version 2.0.1, using the data published by Chang et al. in 2020. Power of the study was kept at 95% and margin of error was kept at 0.05%. After written informed consent, participants greater than 25 years of age were included in the study and divided into three groups: DKD (68 patients having diabetic kidney disease), DM (68 patients having Type-II DM but no evidence of onset of DKD) and 68 Healthy controls. To avoid confounding, participants in each group were age and sex matched. A Type-II diabetic was considered to have diabetic kidney disease if he/she had an elevated albuminuria of >300 mg/24 hours, or an albumin-to-creatinine ratio (ACR) > 300 mg/g (macroalbuminuria), confirmed in at least two of three samples, in the absence of signs of other forms of renal disease. For inclusion of Type-II diabetics the following criteria was considered: fasting plasma glucose level (FPG) > 126mg/dl, or two hours plasma glucose level > 200 mg/dl during oral glucose tolerance test (OGT), or plasma level of haemoglobin A1C (A1C) > 6.5 % or in a person with classic symptoms of hyperglycaemia and hyperglycaemic crises a random plasma glucose level of > 200 mg/dl. Age and sex matched healthy controls having fasting plasma level of glucose (< 100 mg/dl) having no albuminuria (< 30 mg/dl) and no other kidney or endocrine disease were obtained from CMH Lahore and Surgimed Hospital / Lahore Medical and Dental College, Lahore after obtaining relevant ethical approvals.

Blood samples were drawn after aseptic measures, and a midstream urine sample was also obtained. Biochemical analysis was performed by spectrophotometry using standardized kits as per manufacturers protocols to determine the following parameters: fasting plasma glucose, fasting serum insulin, glycated haemoglobin, lipid profile, urinary albumin, urinary creatinine and urinary albumin to creatinine ratio. HOMA-IR and eGFR were calculated after obtaining the biochemical results. RNA extraction from whole blood was performed by using Trizol reagent as per manufacturers protocol and was used for cDNA synthesis using commercially available kit (RevertAid First Strand cDNA Synthesis kit by Thermo Scientific (catalogue number K1622). Real time PCR was used to determine the relative expression of MEG3 lnc-RNA. The following sequence of primers was used: 5′-GGA AGG GAC CTC GAA TGT GG-3′(forward) and 5′-CTG TCC CGT GGG AAT AGG TG-3′(reverse). GAPDH was used as a house keeping gene having the primer sequence 5-TGA CTT CAA CAG CGA CAC CCA-3′(forward) and 5-CAC CCT GTT GCT GTA GCC AAA-3′(reverse). The RNA expression was calculated by using the 2^-∆∆ct^ method. Relative RNA expression was expressed as fold change (fc).

### Statistical analysis:

Data was analyzed by using SPSS version 26. The normality of the numeric data such as age, anthropometric variables, lipid profile, renal function test and RNA expression was explored by using the Shapiro Wilk test. Consequently, non-parametric tests were utilized on the current data since it was non-normally distributed. Thus, Kruskal Wallis test was employed for comparison of three groups while between the group comparisons were made by Mann Whiteny U test. The overall efficacy for three group comparison was calculated by the eta squared method while the efficacy of between the group comparisons was made by rank-biserial correlation coefficient (*r)* method*. C*orrelation between MEG3 lnc-RNA expression and biochemical parameters was measured by Spearman’s Rho Correlation. A p-value of < 0.05 was taken as statistically significant.

## RESULTS

The study comprised 204 participants, 68 in each group having DKD, Type-II DM and healthy controls. There were 128 males and 76 females in the study. The participants had a median age of 55 years (IQR= 50-62), 51 years (IQR= 45-60) and 52 years (IQR= 45-60) in the three groups respectively. The median duration of DKD was 3(1.25-6) years in the patients suffering from DKD, whereas the median duration of Type-II DM was 12(10-16) years in DKD group and 5(2-10) years in the DM group. Fasting plasma glucose, glycated hemoglobin, fasting insulin, HOMA-IR and lipid profile were found to be the highest in the diabetic group, whereas the markers of diabetic kidney disease were severely deranged in the DKD group, having high values of serum urea, serum creatinine and urinary albumin with lowest values of estimated glomerular filtration rate and urinary creatinine among the three groups. The biochemical parameters of the study participants are given in Table-I.

**Table-I:** Biochemical parameters of the study participants.

Parameter (units)	DKD n=68 Median (IQR)	DM n=68 Median (IQR)	Healthy n=68 Median (IQR)
Fasting plasma glucose (mg/dl)	169 (141-260)	174 (127.25-230.5)	96 (93-98)
Glycated haemoglobin (%)	6.9 (5.8-8.35)	8.45 (6.9-10.15)	4.8 (4.5-5.0)
Fasting insulin (IU)	4.59 (2.94-9.23)	16.73 (8.11-21.74)	3.97 (2.01-5.96)
HOMA IR	2.17 (1.22-5.22)	6.36 (3.14-15.68)	0.92 (0.48-1.38)
Total lipid (TL) (mg/dl)	641 (578-708)	755.5 (668-816.5)	651.10 (622.4-675.2)
Serum total cholesterol (TC) (mg/dl)	145 (129.75-169.75)	179.5 (164.00-205.75)	164.5 (155-179.75)
Serum high density lipoprotein (HDL) (mg/dl)	40 (37.25-42.00)	41.50 (40-43)	41.20 (41-43.75)
Serum low density lipoprotein (LDL) (mg/dl)	77 (68.75-96.05)	99.10 (89.85-117.35)	96.4 (87.4-107.00)
Serum triglyceride (mg/dl) (TG)	144.5 (111.25-169.00)	174 (140.25-223.5)	124 (110.25-132)
Very low-density lipoprotein (VLDL) (mg/dl)	28.9 (22.25-33.80)	34.8 (28.05-44.7)	23.7 (20.85-26.55)
Serum urea (mg/dl)	131.5 (94.7-177.00)	29.00 (22.5-34.00)	22.00 (18.25-25.75)
Serum creatinine (mg/dl)	6.5 (4.15-8.32)	0.75 (0.60-0.90)	0.70 (0.60-0.80)
Estimated glomerular filtration rate CKD EPI Pak (ml/min/1.73m^2^)	7.50 (6.00-15.75)	103.50 (92.00-114.00)	114.00 (102.0-121.0)
Estimated glomerular filtration rate (MDRD) (ml/min/1.73m^2^)	8.00 (6.00-15.50)	103.5 (85.25-119.00)	111.50 (95.0-126.0)
Urinary albumin (mg/dl)	145.5 (108.0-190.75)	31.95 (25.15-41.90)	9.30 (2.12-13.35)
Urinary creatinine (mg/dl)	96.0 (82.00-120.75)	191.50 (145.00-238.75)	631.50 (355.0-750.0)
Albumin to creatinine ratio	1424.66 (1144.07-1894.5)	197.16 (137.24-254.8)	15.06 (5.90-19.65)

The median (IQR) fold change of MEG3 lnc-RNA expression was slightly higher in the DKD group as compared to the DM group i.e., 3.38(1.22-3.83) and 2.22(0.49-3.12) respectively which was significantly higher (*p*-value= 0.002) from controls having the median (IQR) values of 1.97(1.17-1.97) with a moderate effect size of η^2^_H_= 0.06. Pairwise analysis further revealed that MEG3 lnc-RNA expression was significantly different between DKD and healthy groups (*p-*value= 0.001), and between DKD and DM groups (*p*-value = 0.049) with effect sizes of 3.04 and 2.07 respectively. On the contrary, the difference between the healthy and DM group was statistically insignificant (*p*-value= 0.783) having an effect size of 0.97 as evident from the Table-II.

**Table-II:** Comparison of MEG3 lnc-RNA expression among the study groups.

	Group	Median (IQR)	p-value	Effect size η^2^_H_	Pair wise comparison	Test statistic	p-value	Effect size (r)
MEG3 (fc)	DKD n=68	3.83 (1.22-3.83)	0.002*	0.06	DKD-DM	1.61	0.049*	2.07
	DM n=68	2.22 (0.49-3.12)			DKD-Healthy	1.86	0.001*	3.04
	Healthy n= 68	1.97 (1.17-1.97)			DM-Healthy	0.25	0.783	0.97

* Statistically significant (*p*= <0.05; Group means compared by using Kruskal Wallis test; Post Hoc analysis by Mann Whitney U test.

The Spearman Rho correlation coefficient was used to determine the correlation between MEG3 lnc-RNA expression and the biochemical parameters in the three groups. Meg3 lnc-RNA expression showed a significant positive correlation with fasting insulin in the DKD group, whereas it showed a significant negative correlation with fasting insulin in the DM group. It also depicted a weak but significant negative correlation with serum very low-density lipoproteins in the healthy group. Regarding the urinary parameters, only significant negative correlation was shown between MEG3 lnc-RNA expression and urinary creatinine in the DKD group. These results are shown in Table-III.

**Table-III:** Correlation of MEG3 lnc-RNA expression with biochemical parameters in DKD DM and healthy groups.

Parameter	Correlation coefficient p-value	Correlation coefficient p-value	Correlation coefficient p-value
	DKD n=68	DM n=68	Healthy n=68
Fasting plasma glucose mg/dl	ρ= -0.072	ρ=0.002	ρ=0.065
	*p=* 0.562	*p=*0.988	*p=*0.597
Glycated haemoglobin %	ρ=0.059	ρ=0.012	ρ=-0.193
	*p=*0.632	*p=*0.921	*p=*0.115
Fasting Insulin IU/L	ρ=0.266	ρ=-0.369	ρ=-0.196
	*p=*0.028*	*p=*0.002*	*p=*0.108
HOMA IR	ρ=-0.072	ρ=0.002	ρ=0.065
	*p=*0.562	*p=*0.988	*p=*0.597
Serum total cholesterol mg/dl	ρ=0.066	ρ=0.013	ρ=-0.071
	*p=*0.592	*p=*0.915	*p=*0.567
Serum HDL mg/dl	ρ=-0.068	ρ=-0.003	ρ=0.008
	*p=*0.583	*p=*0.981	*p=*0.947
Serum LDL mg/dl	ρ=0.057	ρ=-0.006	ρ=-0.197
	*p=*0.647	*p=*0.961	*p=*0.108
Serum TG mg/dl	ρ=0.072	ρ=-0.006	ρ=-0.107
	*p=*0.558	*p=*0.961	*p=*0.386
Serum VLDL mg/dl	ρ=0.072	ρ=-0.015	ρ=-0.294
	*p=*0.558	*p=*0.905	*p=*0.015*
Serum Urea (mg/dl)	ρ=0.039	ρ=-0.076	ρ=-0.042
	*p=*0.752	*p=*0.537	*p=*0.734
Serum creatinine (mg/dl)	ρ=0.033	ρ=-0.056	ρ=0.040
	*p=*0.790	*p=*0.650	*p=*0.748
Estimated glomerular filtration rate CKD EPI	ρ=-0.134	ρ=0.082	ρ=0.021
	*p=*0.274	*p=*0.506	*p=*0.864
Estimated GRF MDRD	ρ=-0.131	ρ=0.085	ρ=0.088
	*p=*0.288	*p=*0.492	*p=*0.477
Urinary albumin (mg/dl)	ρ=0.014	ρ=-0.029	ρ=-0.059
	*p=*0.909	*p=*0.815	*p=*0.631
Urinary creatinine (mg/dl)	ρ=-0.275	ρ=-0.058	ρ=0.096
	*p=*0.023*	*p=*0.640	*p=*0.438
Albumin to creatinine ratio	ρ=0.156	ρ=-0.028	ρ=-0.145
	*p=*0.205	*p=*0.819	*p=*0.238

* statistically significant (*p=* < 0.05) ρ = Spearman Rho Coefficient

## DISCUSSION

The current study provides newer insights into the role of long noncoding RNAs such as MEG3 in the diabetic patients of our local population, with and without microvascular complications such as DKD. The findings highlight the potential role of MEG3 in the pathogenesis of DKD and its progression, and reveal significant correlations between MEG3 expression and other biochemical parameters, paving the way for future research in the therapeutic role of lnc-RNAs in the local diabetic population.^16^ The key pathological findings include the possible role of this lnc-RNA in the progression of inflammatory response, enhanced fibrosis and podocyte injury.

The results of the study showed a statistically significant difference in the expression of MEG3 lnc-RNA across the three groups. The pairwise comparison showed that the expression of MEG3 lnc-RNA was higher in the DKD group as compared to the DM and with the healthy group. There was no significant difference between the expression of this RNA between the DM and healthy groups. The results of this study are in line with the experiments conducted by Chinese researchers in 2019.^17^ Their study also showed a higher expression level of MEG3 lnc-RNA in diabetic animal models. It was postulated that MEG3 promotes the inflammatory response, leading to enhanced fibrosis^18^ in the renal tissue, ultimately causing an inflammatory response.

The researchers proposed that this is done by the involvement of other non-coding RNAs such as the miR-181a which mediates the inflammatory signaling process through the involvement of the toll like receptor (TLR). Another study conducted in the by Li et al. in 2019 also found similar results regarding the expression of MEG3 lnc-RNA in the serum of patients of DKD. It was demonstrated that not only the expression of MEG3 was 4-fold upregulated in the serum, it was also highly expressed in patients with different stages of diabetic kidney disease, suggesting that this lnc-RNA plays a role not only in disease development, but also in disease progression. This observation was followed by an experiment to knock down the MEG3 mediated fibrosis and apoptosis, to validate the contribution of MEG3 in the progression of DKD. MEG3 knockdown resulted in reduced synthesis of collagen IV and fibronectin, thus establishing the role of MEG3 lnc-RNA in fibrosis.^19^

In contrast, the study conducted by Che et al. in 2019 reported a reduced expression of MEG3 lnc-RNA in cultured podocytes in streptozocin-induced diabetic rats. This study showed that in animal models, a high glucose environment led to suppression of MEG3 lnc-RNA expression, causing activation of the β-catenin signaling pathways, causing podocyte injury. When the β-catenin pathway was suppressed, over expression of MEG3 lnc-RNA and the high glucose induced podocyte injury was attenuated.^20^ The expression of MEG3 lnc-RNA was upregulated in Type-II diabetes mellitus with and without microvascular complications. However, this was only a small increase in the expression of MEG3 (1.28 times) as compared to the control group.

Chang et al. also reported a positive correlation between MEG3 lnc-RNA and plasma glucose and HbA1c levels.^21^ In the current study, however, no significant correlation was seen between MEG3 lnc-RNA and these parameters, but there was a significant positive correlation with fasting insulin levels in the DKD group, and a statistically significant negative correlation with fasting insulin levels in the DM group. This trend of a positive correlation of MEG3 expression with fasting insulin in DKD may indicate a compensatory mechanism to counteract insulin resistance in DKD. In contrast, the negative correlation of MEG3 expression with fasting insulin in the DM group suggests that MEG3 might play a potentially different role in diabetes before onset of DKD, by regulating glucose metabolism and insulin sensitivity. Contrasting results of the current study were also observed as compared to a study conducted by Elwi et al. in 2022, who reported a significant negative correlation between MEG3 lnc-RNA and HbA1c.^22^

There was a significant negative correlation between MEG3 lnc-RNA expression and serum very low-density lipoproteins in the healthy group, which is supported by the study of Daneshmoghadam et al. in 2020. It was hypothesized that MEG3 lnc-RNA plays a pivotal role in the activity of adipogenesis and lipogenesis by affecting the enzymes such as fatty acid synthase and acetyl-CoA carboxylase.^23^

The current study also showed a significant negative correlation between MEG3 lnc-RNA expression and urinary creatinine levels in the DKD group. This was in contrast to the data reported in 2024, which showed a significant positive correlation between MEG3 lnc-RNA expression and serum creatinine levels in DKD.^24^

Thus, it was concluded that MEG3 lnc-RNA is involved in the pathogenesis of diabetic kidney disease through various inflammatory and cell signaling pathways; however, the expression of MEG3 lnc-RNA was higher or lower depending on the source of RNA isolation such as serum, peripheral blood mononuclear cells, or cultured podocytes.^22^ The exact mechanism of MEG3 lnc-RNA expression in response to high glucose remains elusive, however, it has been hypothesized that high glucose in the local environment enhances the transcriptional activity of the genes leading to over expression of this lnc-RNA, which in turn plays its role in various cells and pathways involved in the pathogenesis of diabetic kidney disease.^25^ The current study provides novelty in terms of exploration of lnc-RNA expression in the local population of Lahore, and explores this expression with a comprehensive approach to the disease at various stages of disease development. It provides a clinical relevance related to a possible earlier diagnosis of DKD and its progression from less severe to severe forms. Further research is needed to highlight the therapeutic role of MEG3 lnc-RNA in prevention of DKD in Type-II diabetics, and for reversal of high glucose mediated injury.

### Limitations

This was a cross-sectional study with no follow-up. As a result, the causal relationship between lnc-RNA expression and levels of serum and urinary biochemical parameters cannot be established.

## CONCLUSION

MEG3 lnc-RNA is expressed more in patients suffering from DKD as compared to people with diabetes without DKD and healthy controls. There are weak correlations between MEG3 lnc-RNA expression and markers of disease severity in DKD and type-II DM. This may be a key link to establish the role of MEG3 lnc-RNA as a possible diagnostic marker for early detection of disease as well as a marker for understanding disease progression. Future research may explore the role of lnc-RNAs as therapeutic targets.

### Authors’ Contributions:

**HSK:** Conception of idea, study design, sampling, data entry and analysis, scientific write up, results, referencing and proof reading of the manuscript.

**UZ:** Co-supervision of the project, recruitment and sampling of diabetics and healthy controls, scientific writing,

**WA:** Co-supervision of the project, diagnosis and recruitment of DKD patients.

**SK:** Overall supervision of the project, RNA isolation, RT-PCR and analysis of RNA expression, final draft of manuscript.

All authors have read and approved the final version and they are also responsible for accuracy and integrity of work.

## References

[1] WHO Diabetes Mellitus.

[2] IDF (2025). IDF Diabetes Atlas 2025 [Online]. Brussels, Belgium.

[3] Sugahara M, Pak WLW, Tanaka T, Tang SC, Nangaku M (2021). Update on diagnosis, pathophysiology, and management of diabetic kidney disease. Nephrology..

[4] Jabeen WM, Jahangir B, Khilji S, Aslam A (2023). Association of triglyceride glucose index and triglyceride HDL ratio with glucose levels, microvascular and macrovascular complications in Diabetes Mellitus Type-2. Pak J Med Sci..

[5] Yamazaki T, Mimura I, Tanaka T, Nangaku M (2021). Treatment of diabetic kidney disease:current and future. Diabetes Metab J..

[6] Sandholm N, Dahlstrom EH, Groop PH (2023). Genetic and epigenetic background of diabetic kidney disease. Front Endocrinol (Lausanne)..

[7] Al-Rugeebah A, Alanazi M, Parine NR (2019). MEG3:an oncogenic long non-coding RNA in different cancers. Path Oncol Res.

[8] Hamilton S, de Cabo R, Bernier M (2020). Maternally expressed gene 3 in metabolic programming. Biochimica et Biophysica Acta (BBA)-Gene Regulatory Mechanisms..

[9] Hu X, Zeng F, Chen Z, Hu K, Chen Q, Xia Y (2025). Knockdown of LncRNA MEG3 promotes damage of vascular endothelial cells induced by vibration. Sci Rep..

[10] Luo Q, Xia X, Luo Q, Qiu Y, Dong L, Zhao C (2022). Long noncoding RNA MEG3-205/Let-7a/MyD88 axis promotes renal inflammation and fibrosis in diabetic nephropathy. Kidney Dis..

[11] Chu P-M, Yu C-C, Tsai K-L, Hsieh P-L (2022). Regulation of oxidative stress by long non-coding RNAs in vascular complications of diabetes. Life..

[12] Rong L, Xue H, Hao J, Liu J, Xu H (2024). Long non-coding RNA MEG3 silencing weakens high glucose-induced mesangial cell injury by decreasing LIN28B expression by sponging and sequestering miR-23c. Kidney Res Clin Pract..

[13] Li M-K, Liu L-X, Zhang W-Y, Zhan H-L, Chen R-P, Feng J-L (2020). Long non-coding RNA MEG3 suppresses epithelial-to-mesenchymal transition by inhibiting the PSAT1-dependent GSK-3β/Snail signaling pathway in esophageal squamous cell carcinoma. Oncol Rep..

[14] Khan FA, Fatima SS, Khan GM, Shahid S (2019). Evaluation of kidney injury molecule-1 as a disease progression biomarker in diabetic nephropathy. Pak J Med Sci..

[15] Huang S-F, Peng X-F, Jiang L, Hu CY, Ye W-C (2021). LncRNAs as therapeutic targets and potential biomarkers for lipid-related diseases. Front Pharmacol..

[16] Hossam Abdelmonem B, Kamal LT, Wardy LW, Ragheb M, Hanna MM, Elsharkawy M (2025). Non-coding RNAs:emerging biomarkers and therapeutic targets in cancer and inflammatory diseases. Front Oncol..

[17] Zha F, Qu X, Tang B, Li J, Wang Y, Zheng P (2019). Long non-coding RNA MEG3 promotes fibrosis and inflammatory response in diabetic nephropathy via miR-181a/Egr-1/TLR4 axis. Aging (Albany NY)..

[18] Jiang X (2025). Long noncoding RNA MEG3:an active player in fibrosis. Pharmacol Rep..

[19] Li J, Jiang X, Duan L, Wang W (2019). Long non-coding RNA MEG3 impacts diabetic nephropathy progression through sponging miR-145. Am J Transl Res..

[20] Che X, Deng X, Xie K, Wang Q, Yan J, Shao X (2019). Long noncoding RNA MEG3 suppresses podocyte injury in diabetic nephropathy by inactivating Wnt/beta-catenin signaling. Peer J..

[21] Chang WW, Zhang L, Yao X-m, Chen Y, Zhu L-j, Fang Z-m (2020). Upregulation of long non-coding RNA MEG3 in type 2 diabetes mellitus complicated with vascular disease:a case–control study. Mol Cell Biochem..

[22] Elwi HM, El-Din SS, Rashed L, Sadek NA, Medhat E (2022). Regulatory Role of Long Non-Coding RNA “Maternally-Expressed Gene-3”in Diabetic Nephropathy through Targeting microRNA-21 and Transforming Growth Factor-Beta. Egypt Acad J Bio Sci, Clin Physiol Mol Bio..

[23] Daneshmoghadam J, Omidifar A, Akbari Dilmaghani N, Karimi Z, Emamgholipour S, Shanaki M (2021). The gene expression of long non-coding RNAs (lncRNAs):MEG3 and H19 in adipose tissues from obese women and its association with insulin resistance and obesity indices. J Clin Lab Analysis..

[24] Ting K-H, Yang P-J, Tsai P-Y, Lee C-Y, Yang S-F (2024). Correlations between the long noncoding RNA MEG3 and clinical characteristics for diabetic kidney disease in type 2 diabetes mellitus. Diabetol Metabol Synd..

[25] Xu B-W, Rao Y, Wang L, Chen S-M, Zou S-B (2023). LncRNA MEG3 inhibits renal fibrinoid necrosis of diabetic nephropathy via the MEG3/miR-21/ORAI1 axis. Mol Bio Rep..

